# Extraction process and antioxidant and antimicrobial activities of total flavonoids from *Broussonetia papyrifera* leaves

**DOI:** 10.1038/s41598-025-30074-6

**Published:** 2025-12-02

**Authors:** Xiaoqiang Huang, An Jia, Shunhe Liu, Hongchang Zhao, Cheng Ding, Chunsheng Yan

**Affiliations:** 1 School of Medicine, Huanghe Science & Technology University, Zhengzhou, China; 2 School of Pharmacy, Henan University, Kaifeng, China

**Keywords:** *Broussonetia papyrifera* leaves, Total flavonoids, Response surface methodology, Extraction process; antioxidant activity, Antibacterial activity, Biochemistry, Biotechnology, Microbiology, Plant sciences

## Abstract

This study developed an efficient extraction process for flavonoids from *Broussonetia papyrifera* leaves using the response surface methodology. The optimized flavonoids exhibited remarkable antioxidant activity, effectively scavenging DPPH, hydroxyl, and ABTS radicals. UPLC-MS/MS analysis preliminarily identified numerous flavonoid compounds in the extract.. The extract also demonstrated potent broad-spectrum antibacterial efficacy against *Staphylococcus aureus, Escherichia coli,* and *Candida albicans*. These findings indicate that the extracted flavonoids may serve as natural antioxidants and antimicrobials in the food, pharmaceuticals, and cosmetics industries.

## Introduction

*Broussonetia papyrifera* (L.) L’Hér. ex Vent. is native to China and the subtropical regions of Southeast and East Asia^1^. Its roots, bark, and fruits have long been used in traditional Chinese medicine. The species has medicinal, edible, and fodder applications and is valued for its nutrient and bioactive content. Its leaves, rich in amino acids, represent a valuable unconventional feed source for animals^2^. The plant also contains polysaccharides, proteins, flavonoids, phenylpropanoids, and other polyphenols^3^. In traditional Chinese medicine, dried twigs, leaves, and roots are prescribed for diuresis, tonification, and edema reduction^4^.

Flavonoids, a major class of natural polyphenols, include quercetin, kaempferol, catechin, luteolin, and apigenin^5^. They have been reported to slow aging, alleviate oxidative stress and inflammation, protect the blood–brain barrier, and reduce the risk of cardiovascular disease, immune disease, cancer, and neurodegenerative diseases such as Alzheimer’s disease^6–8^. In animal husbandry, the use of flavonoids has received increasing attention. They are considered potential green feed additives because of their antibacterial, antioxidant, anti-inflammatory, immunomodulatory, and other biological properties, which may reduce the use of traditional antibiotics and improve animal health and production performance^9,10^. To efficiently extract flavonoids, modern technologies such as ultrasound-assisted extraction, microwave-assisted extraction, and enzymatic extraction have been widely applied, substantially improving extraction efficiency and product purity^11,12^.

The traditional extraction methods of flavonoids include solvent extraction, crystallization, microfluidic technology, vacuum extraction, Soxhlet extraction and so on. However, these methods have problems such as long extraction time, expensive extraction equipment, low extraction efficiency, and the need for high purity solvents. Ultrasonic assisted extraction technology is to quickly enter the solvent under ultrasonic action based on the presence, polarity and solubility of other active ingredients in the substance to obtain a multi-component mixture, and then obtain the active substance monomer through appropriate separation and purification techniques. At present, this method has been widely used in the extraction and separation of active ingredients in natural products. Combining ultrasonic waves with traditional solvent extraction has the advantages of reducing extraction time, targeted heating, reducing solvent consumption, and high extraction rate, making it an effective method for extracting total flavonoids^13^. Currently, ultrasound has been used to extract a variety of plant flavonoids^14,15^. The study found that compared with the traditional Soxhlet extraction method, ultrasonic assisted extraction of total flavonoids can shorten the extraction time and improve the yield^16^. Ultrasonic assisted extraction technology can effectively avoid the damage of active ingredients by high temperature, and its extraction efficiency is mainly affected by key parameters such as ultrasonic frequency, time, and temperature. Therefore, determining appropriate ultrasonic extraction parameters is key to improving extraction efficiency. Meanwhile, the integration of ultrasonic technology with other emerging technologies will become a research hotspot. With the continuous innovation of scientific research technologies, ultrasonic extraction technology will demonstrate broader application prospects in fields such as food processing, pharmaceutical R&D, and chemical production^17^.

Although previous studies have examined the antioxidant activity of *B. papyrifera* leaves and the extraction process of total flavonoids, effective extraction and comprehensive evaluation remain at an exploratory stage. In particular, optimization of extraction parameters and systematic evaluation of the antioxidant and antibacterial potential of flavonoids to maximize their yield have not been fully resolved.

## Materials and methods

### Materials

*B. papyrifera* leaves were collected from the campus of Yellow River Institute of Science and Technology in September 2022. Botanical identification and confirmation of the leaves as being *Broussonetia papyrifera* (L.) L’Hér. ex Vent was performed by Professor Wang Li (Department of Pharmacy, School of Medicine, Yellow River Institute of Science and Technology). This species is currently preserved in the Herbarium of Huanghe University of Science and Technology (designation No. 20220622). Rutin (standard compound), antioxidant assay reagents, and the microbial strains *Escherichia coli* (ATCC 25,922), *Staphylococcus aureus* (ATCC 25,923), and *Candida albicans* (ATCC 10,231) were provided by the Microbiology Laboratory of the Medical College of the same institution.

### Determination of the total flavonoid content in *B. papyrifera* leaves

The total flavonoid content in the leaves was determined by NaNO_2_–Al(NO_3_)_3_ colorimetry^18,19^. The calibration curve yielded a linear regression equation Y = 9.7522X—0.0128 (R^2^ = 0.9993), displaying linearity from 0 to 0.06 mg/mL. The total flavonoid yield was calculated using the following formula:

$${\text{Total}}\;{\text{flavonoids}}\;{\text{yield/}}\% = \frac{{C \times V \times N}}{{M \times 1000}} \times 100$$

Note: C is the total flavonoid concentration (expressed in mg/mL); V is the fixed volume (expressed in mL); M is the mass (expressed in g); N is the dilution multiple.

### Single-factor experiment for the extraction of total flavonoids in *B. papyrifera* leaves

The collected fresh *B. papyrifera* leaves were dried in an oven, crushed, and sieved through a 20-mesh sieve to obtain *B. papyrifera* leaf powder and saved. The extraction yield of total flavonoids from *B. papyrifera* leaves was evaluated by using a single-factor experimental design as described elsewhere^14,15^. A single-factor experimental design was employed to investigate the effects of extraction time (i.e., 10, 20, 30, 40, 50 min), extraction temperature (i.e., 30, 40, 50, 60, 70 °C), solid-to-liquid ratio (i.e., 1:10, 1:20, 1:30, 1:40, 1:50 g/mL), and ethanol volume fraction (i.e., 50, 60, 70, 80, 90%). Each factor was tested independently across the specified levels.

### Response surface method optimization experiment

Based on the results of the single-factor experiments, a response surface optimization experiment was designed using the Design-Expert 10.0 software. The total flavonoid yield served as the response variable. The experimental design matrix is presented in Table 1.

**Table 1:** Factors and Levels of Experimental Design of Response Surface.

Level	Factor			
	Extraction time/min	Extraction temperature/^°^C	Material-liquid ratio/(g/mL)	Volume fraction of ethanol/%
-1	20	40	1:20	70
0	30	50	1:30	80
1	40	60	1:40	90

### Identification of flavonoid constituents

To comprehensively characterize the chemical profile of the flavonoids present in the *B. papyrifera* leaves extract, a qualitative analysis was performed using ultra-performance liquid chromatography coupled with tandem high-resolution mass spectrometry (UPLC-Orbitrap-MS).

The collected fresh *B. papyrifera* leaves were dried in an oven, crushed and sieved through a 20-mesh sieve to obtain *B. papyrifera* leaf powder. The powdered *B. papyrifera* leaves were extracted with 83% ethanol at a solid-to-liquid ratio of 1:35 (g/mL) using ultrasound-assisted extraction at 54.7 °C for 24.3 min. The resulting filtrate was collected, and the solvent was removed under reduced pressure. The remaining extract was used for subsequent experiments.

The extracted sample were analyzed using a UPLC-Orbitrap-MS system (UPLC, Vanquish; MS, HFX). UPLC conditions: The analytical conditions were as follows, UPLC: column, Waters HSS T3(100 × 2.1 mm, 1.8 μm); column temperature, 40 °C; flow rate, 0.3 mL/min; injection volume, 2 μL; solvent system, phase A were Milli-Q water (0.1% formic acid), phase B were acetonitrile (0.1% formic acid); gradient program, 0 min phase A/phase B (100:0, v/v), 1 min phase A/phase B (100:0, v/v), 12 min phase A/phase B (5:95, v/v), 13 min phase A/phase B (5:95, v/v), 13.1 min phase A/phase B (100:0, v/v), 17 min phase A/phase B (100:0, v/v). MS conditions: HRMS data were recorded on a Q Exactive HFX Hybrid Quadrupole Orbitrap mass spectrometer equipped with a heated ESI source (Thermo Fisher Scientific) utilizing the Full-ms-ddMS2 acquisition methods. The ESI source parameters were set as follows: sheath gas pressure, 40 arb; aux gas pressure, 10 arb; spray voltage, + 3000 v/-2800 v; temperature, 350℃; and ion transport tube temperature, 320℃. The scanning range of the primary mass spectrometry was (scan m/z range) 70–1050 Da, with a primary resolution of 70,000 and secondary resolution of 17,500.

### External antioxidant experiment

#### Ability to remove DPPH-free radicals

Based on the methods of Zhang et al.^20,21^ with some modifications, 3 mL aliquots of the total flavonoid solution (derived from *B. papyrifera* leaves) at different mass concentrations were accurately transferred into test tubes, to which 3 mL of the DPPH solution was added. The mixtures were incubated in the dark for 30 min, and the absorbance (A₁) was measured at 517 nm. Using the same procedure, the absorbance values A_2_ and A_0_ were determined. Vitamin C (VC) was used as a positive control. The IC_50_ value was calculated as follows:

$${\text{The}}\;{\text{clearance}}\;{\text{of}}\;DPPH/\% = \left[ {1 - \left( {A1 - A2} \right)} \right]A0 \times 100$$

#### Ability to remove hydroxyl-free radicals

Based on the methods of Zhao et al.^20,21^ with modifications, 2 mL aliquots of each different mass concentration of the total flavonoid solution were accurately transferred into test tubes, to which 2 mL each of FeSO₄ solution, salicylic acid–ethanol solution, and H_2_O_2_ solution were added sequentially. The mixture was mixed well and allowed to react for 30 min. The absorbance (A₁) was measured at 510 nm. Using the same procedure, the absorbance values A_2_ and A_0_ were determined. VC was used as a positive control. The IC_50_ value was calculated as follows:

$${\text{The}}\;{\text{clearance}}\;{\text{of}}\;{\text{hydroxyl}}\;{\text{radical/}}\% = \left[ {1 - \left( {A1 - A2} \right)} \right]A0 \times 100$$

#### The ability to remove ABTS-free radicals

Based on the method of Chai et al.^20,21^ with some modifications, ABTS and K_2_S_2_O₈ were accurately weighed into a 25-mL volumetric flask. The reagents were mixed at a volume ratio of 1:1, protected from light, and stored at room temperature for 12–16 h to prepare the ABTS stock solution. Subsequently, the stock solution was diluted with anhydrous ethanol until its absorbance at 734 nm reached 0.7 ± 0.02, yielding the ABTS working solution.

Next, 1 mL aliquots of the total flavonoid solution (derived from *B. papyrifera* leaves) at different mass concentrations were accurately transferred. To each aliquot, 2.0 mL of the ABTS-working solution was added. The solutions were mixed well and incubated in the dark at room temperature for 10 min. The absorbance at 734 nm (A₁) was measured. Using the same procedure, the absorbance values A_2_ and A_0_ were determined. VC served as a positive control. The IC_50_ value was calculated as follows:

$${\text{The clearance of ABTS}}/{{\% }} = \left[ {1 - \left( {A1 - A2} \right)} \right]A0 \times 100$$

### In vitro bacteriostatic experiment

#### Bacterial solution preparation

Each of the three bacterial strains was inoculated into nutrient broth and cultured for 24 h at 37 °C with 120 rpm agitation.

### Determination of MIC

A modified method based on the literature^22,23^ was used to determine the MIC of total leaf flavonoids against *S. aureus*, *E. coli*, and *C. albicans* using serial dilution. Briefly, eight test tubes were prepared, each containing 3 mL of nutrient broth. To tube 1, 1 mL of the sample solution (100 mg/mL) was added and mixed thoroughly. Then, 1 mL was transferred from tube 1 to tube 2, mixed well, and this serial dilution was repeated sequentially through tube 5. Tube 6 served as a negative control (growth control), tube 7 as a positive control (penicillin), and tube 8 as a sterility control (nutrient broth only). Subsequently, 500 μL of the bacterial suspension was added to each of the tubes 1–7. After 24 h of incubation at 37 °C, the MIC was recorded as the lowest extract concentration, showing no turbidity in the culture medium.

### Statistical analysis

All experiments were repeated three times. Data were processed and visualized using GraphPad Prism 5 and Design-Expert 10 software. One-way analysis of variance (ANOVA) was performed with a 95% confidence level.

## Results and analysis

### Single-factor test results

#### Effect of extraction time on total flavonoid yield

**Fig. 1:**
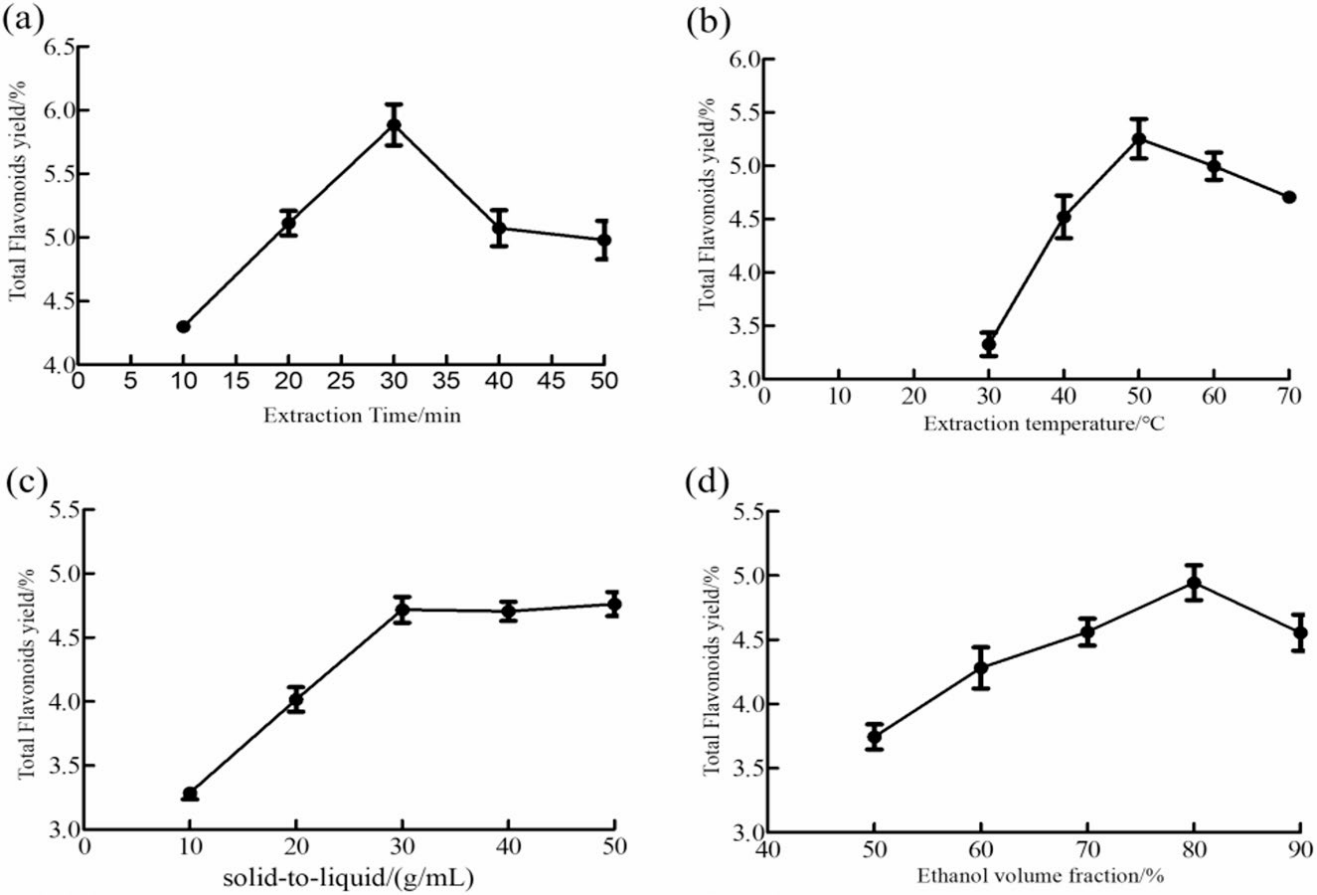
Effect of extraction time (**a**), extraction temperature (**b**), solid-to-liquid ratio (**c**), and ethanol volume fraction (**d**) on the total flavonoid yield.

Figure 1a shows that the total flavonoid extraction rate increased from 10 to 30 min but decreased between 30 and 50 min. The maximum yield (5.88%) was observed at 30 min. This trend could be attributed to the initial absorption of ultrasonic energy by leaf cells, which elevates intracellular temperatures and facilitates flavonoid dissolution. However, prolonged ultrasound exposure (> 30 min) likely degrades flavonoids, reducing the overall yield^24^. Consequently, ultrasonic extraction times of 20, 30, and 40 min were selected for response surface optimization.

### Effect of extraction temperature on total flavonoid yield

Figure 1b shows that total flavonoid yield increased at 30 °C–50 °C but decreased at 50 °C–70 °C, peaking at 50 °C (5.25%). This trend suggests that within a moderate temperature range, thermal acceleration of molecular mobility enhances flavonoid dissolution from leaf tissues. However, elevated temperatures (> 50 °C) may reduce yield due to thermal degradation of flavonoid compounds and volatilization of ethanol, which decreases extraction efficiency^25^. Therefore, temperatures of 40 °C, 50 °C, and 60 °C were selected for subsequent response surface optimization.

### Effect of solid-to-liquid ratio on total flavonoid yield

Figure 1c shows that total flavonoid yield increased progressively from solid-to-liquid ratios of 1:10–1:30 g/mL, then stabilized between 1:30 and 1:50 g/mL. The maximum yield (4.65%) occurred at 1:30 g/mL. At lower ratios, limited contact between leaf tissue and solvent restricted flavonoid dissolution, reducing yield. Higher ratios improved solvent–material contact, promoting dissolution until saturation was reached. Beyond this point, further increases in the ratio provided no significant improvement because flavonoids were fully dissolved^26^. Consequently, ratios of 1:20, 1:30, and 1:40 g/mL were selected for subsequent response surface optimization.

### Effect of ethanol volume fraction on total flavonoid yield

Figure 1d indicates that total flavonoid yield increased from 50 to 80% ethanol concentration but decreased between 80 and 90%. The maximum yield (4.95%) was observed at 80% ethanol. At lower ethanol concentrations (< 80%), flavonoid dissolution from leaf material was incomplete. Increasing ethanol concentration improved dissolution and yield. However, above 80%, decreased solution polarity promoted dissolution of nonpolar compounds while reducing the extraction efficiency of highly polar flavonoids, consequently lowering the total yield^27^. Therefore, ethanol concentrations of 70%, 80%, and 90% were selected for subsequent response surface optimization.

### Response surface optimization

#### Experimental results and analysis of the response surface

Based on the single-factor experiment results, a four-factor, three-level response surface methodology design was employed to optimize the total flavonoid extraction from leaf material. The experimental design matrix and corresponding results are presented in Table 2.

**Table 2:** Experimental results of response surface.

Runs	A	B	C	D	Y
	Solid-to-liquid ratio/g/mL	Extraction time/min	Extraction temperature/℃	Ethanol volume fraction/%	Total flavonoid yield/%
1	1	-1	0	0	5.64
2	0	0	− 1	1	4.58
3	1	1	0	0	4.81
4	0	0	0	0	5.56
5	0	0	1	− 1	4.67
6	− 1	0	1	0	4.81
7	1	0	− 1	0	4.45
8	0	1	0	1	4.21
9	− 1	-1	0	0	4.49
10	0	-1	0	− 1	4.39
11	− 1	1	0	0	4.35
12	0	-1	− 1	0	4.56
13	0	-1	0	1	4.91
14	0	1	-1	0	4.57
15	0	1	1	0	4.89
16	0	0	1	1	5.21
17	1	0	0	1	5.23
18	0	-1	1	0	5.44
19	0	0	0	0	5.78
20	0	0	-1	− 1	3.91
21	0	1	0	− 1	4.83
22	1	0	0	− 1	4.93
23	− 1	0	-1	0	3.93
24	− 1	0	0	− 1	4.04
25	− 1	0	0	1	4.87
26	0	0	0	0	5.36
27	0	0	0	0	5.41
28	0	0	0	0	5.51
29	1	0	1	0	5.23

### Analysis of variance and fitting of quadratic multiple regression equations

Quadratic multivariate regression analysis of the data in Table 2 was performed using Design-Expert 10 software. The regression equation is as follows:

Y = 5.5 + 0.32A − 0.15B + 0.35C + 0.19D − 0.17AB − 0.025AC − 0.13AD − 0.14BC − 0.29BD − 0.033CD − 0.37A^2^ − 0.33B^2^ − 0.44C^2^ − 0.50D^2^.

The analysis of the quadratic multivariate regression model is summarized in Table 3.

**Table 3:** Analysis of the variance of the regression model.

Source	The sum of squares	Degree of freedom	Mean square	F value	*P*	Prominence
Model	6.88	14	0.49	9.57	< 0.0001	**
A	1.20	1	1.20	23.42	0.0003	**
B	0.26	1	0.26	5.08	0.0407	*
C	1.51	1	1.51	29.30	< 0.0001	**
D	0.42	1	0.42	8.14	0.0128	*
AB	0.12	1	0.12	2.32	0.1502	NS
AC	2.500E-003	1	2.500E-003	0.049	0.8286	NS
AD	0.070	1	0.070	1.37	0.2619	NS
BC	0.078	1	0.078	1.53	0.2370	NS
BD	0.32	1	0.32	6.32	0.0247	*
CD	4.225E-003	1	4.225E-003	0.082	0.7785	NS
A^2^	0.89	1	0.89	17.35	0.0010	**
B^2^	0.71	1	0.71	13.92	0.0022	**
C^2^	1.24	1	1.24	24.11	0.0002	**
D^2^	1.59	1	1.59	31.03	< 0.0001	**
Residual	0.72	14	0.051			
Lost item	0.61	10	0.061	2.29	0.2204	NS
Pure error	0.11	4	0.027			
Sum total	7.60	28				

*P < 0.05 and **P < 0.01 indicate significant impact.

Table 3 shows that the model has an *F* value of 9.57 and a *P* value of < 0.01, indicating that it is significant. The lack-of-fit term has a P value of 0.2204, indicating that model error is not significant. A (solid-to-liquid ratio), B (extraction time), C (extraction temperature), and D (ethanol volume fraction) significantly affect the total flavonoid yield of structural leaves (*P* < 0.01, *P* < 0.05, *P* < 0.01, *P* < 0.05, respectively). The quadratic terms A^2^, B^2^, C^2^, and D^2^ are significant (*P* < 0.01). The order of influence of each factor is D > C > A > B. These results indicate that the model is suitable for optimizing the extraction process of total flavonoids from leaf material.

### Interaction analysis

**Fig. 2:**
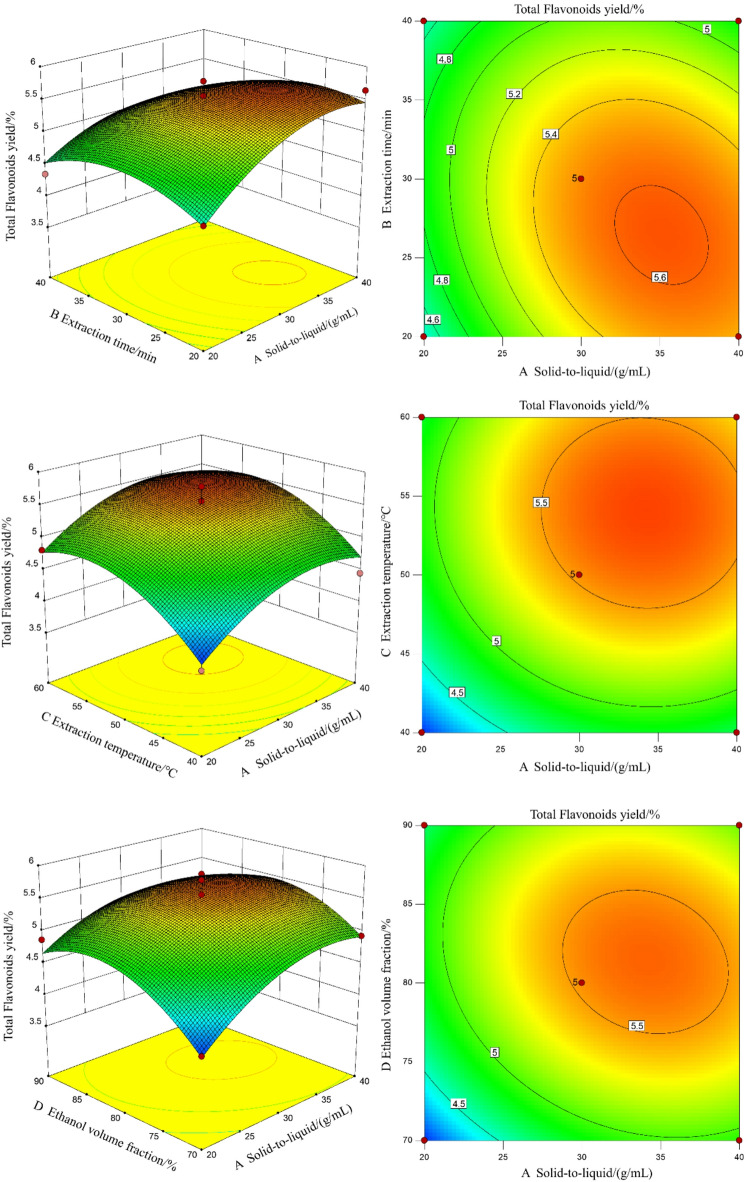
The influence of the interaction of variousdifferent factors on the total flavonoid yield.

Figure 2 illustrates the interactions between factors A–D. The steepness of the response surface reflects the relative influence of each factor on total flavonoid yield from leaf material, whereas contour line density reflects the strength of factor interactions, with elliptical patterns indicating significant interactions^20^. Specifically, the B × D interaction significantly affected flavonoid yield (*P* < 0.05), whereas other factor interactions were not significant.

### Verification test

Based on the response surface model analysis, the optimal extraction conditions were identified as follows: solid-to-liquid ratio, 1:34.95 g/mL; extraction time, 24.34 min; temperature, 54.71 °C; and ethanol concentration, 82.70%. Under these conditions, the predicted total flavonoid yield from leaf material was 5.75%. For practical application, the parameters were adjusted as follows: solid-to-liquid ratio, 1:35 g/mL; extraction time, 24.3 min; temperature, 54.7 °C; and ethanol concentration, 83%. Triplicate verification experiments produced an average total flavonoid content of 5.69% ± 0.04% (mean ± SD), closely matching the predicted value and confirming the model’s predictive validity.

### Analysis of flavonoids by LC–MS/MS

**Fig. 3:**
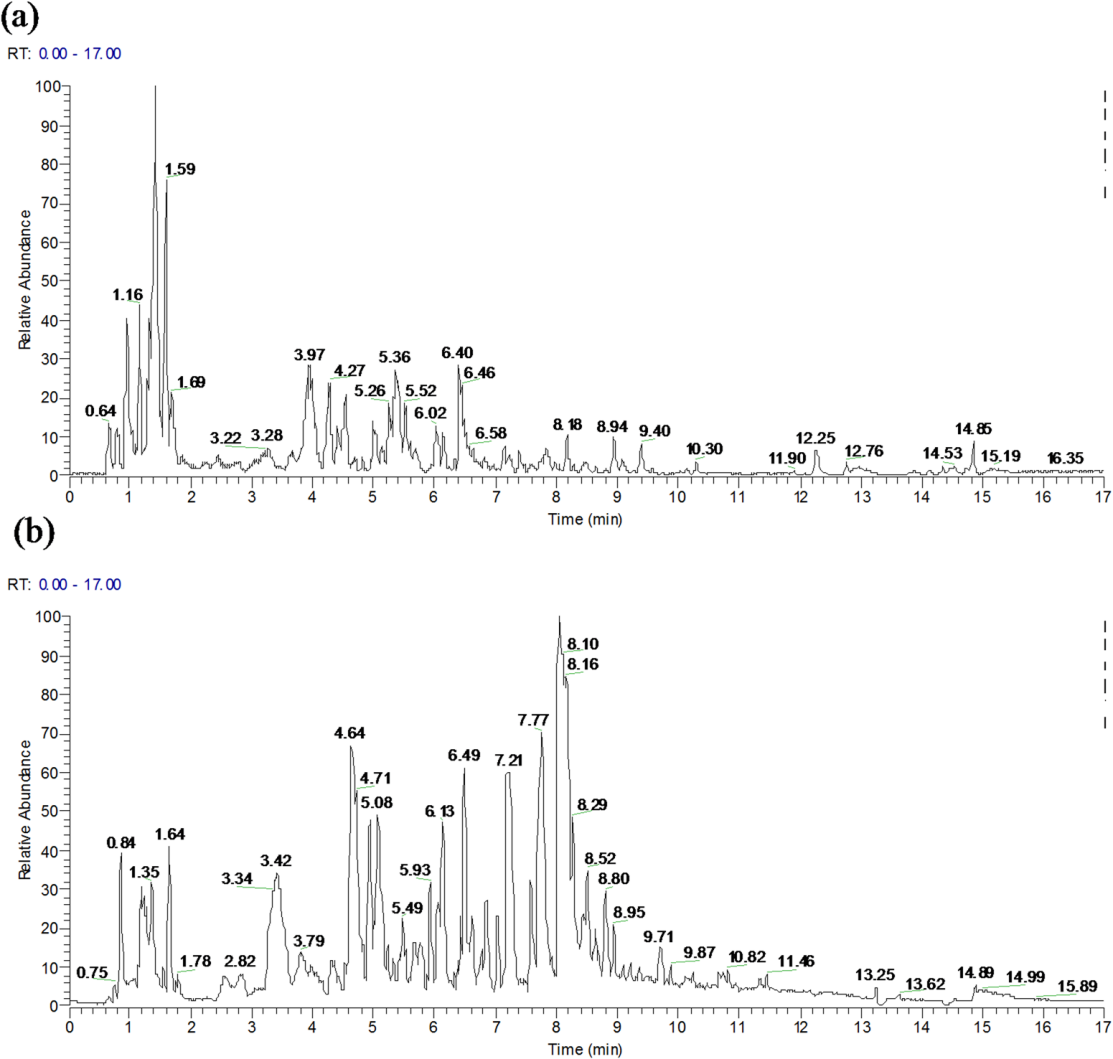
(**a**) and (**b**) represent the total ion chromatogram in postive and negative model of qualitative analysis, respectively.

UPLC-Orbitrap-MS analysis was employed to profile the constituents of the *B. papyrifera* leaf extract (Fig. 3a and b). The raw data were processed using Progenesis QI software (Waters Corporation, Milford, USA), enabling the tentative identification of 101 flavonoid compounds through database comparisons (Table 4).

**Table 4:** Analysis of Flavonoids by LC–MS/MS.

Number	Compound Name	Formula	RT(min)	Calc m/z	Scan Mode
1	Afzelechin 3-O-xyloside	C_20_H_22_O_9_	3.3376	451.1248618	neg
2	Quercetagetin 3,5,7-trimethyl ether	C_18_H_16_O_8_	3.8457	465.1042795	neg
3	Rhamnocitrin	C_16_H_12_O_6_	3.9925	391.0673617	neg
4	Kaempferol 3-glucoside 7-rhamnoside	C_27_H_30_O_15_	4.1566	611.162578	neg
5	Epigallocatechin	C_15_H_14_O_7_	4.5201	337.0931487	neg
6	Kaempferol 3,4’,7-triacetate	C_21_H_16_O_9_	4.5438	517.0991526	neg
7	Taxifolin	C_15_H_12_O_7_	4.6147	303.0511695	neg
8	2’,5,6’,7-Tetraacetoxyflavanone	C_23_H_20_O_10_	4.6957	561.1258193	neg
9	Phloridzin	C_21_H_24_O_10_	4.9575	541.1570876	neg
10	Kaempferol 3-O-vicianoside	C_26_H_28_O_15_	4.9692	611.1628569	neg
11	Kaempferol 3,4’-di-O-glucoside	C_27_H_30_O_16_	4.9979	609.1477069	neg
12	Jaceoside	C_23_H_24_O_12_	4.9979	551.1411313	neg
13	Juglanin	C_20_H_18_O_10_	4.9979	449.1093443	neg
4	Kushenol K	C_26_H_32_O_8_	5.0303	563.2139221	neg
15	Engeletin	C_21_H_22_O_10_	5.0400	479.1199697	neg
16	(-)-Epigallocatechin-3-(3’’-O-methyl) gallate	C_23_H_20_O_11_	5.1567	503.1205013	neg
17	(-)-Epicatechin-3-(3’’-O-methyl) gallate	C_23_H_20_O_10_	5.1760	501.1044214	neg
18	Isoastilbin	C_21_H_22_O_11_	5.1959	449.1092794	neg
19	Cirsimarin	C_23_H_24_O_11_	5.1959	521.1304237	neg
20	Homoorientin	C_21_H_20_O_11_	5.3013	447.0938145	neg
21	Apigenin 7-O-(6’’-O-malonylglucoside)	C_24_H_22_O_13_	5.3357	549.1256207	neg
22	(-)-Epiafzelechin 3-O-gallate	C_22_H_18_O_9_	5.3569	531.1149781	neg
23	Norwogonin-8-O-glucuronide	C_21_H_18_O_11_	5.3865	445.0781551	neg
24	Quercetin 3-O-beta-(6’’-p-coumaroyl)glucopyranosyl(1- > 2)-alpha-L-rhamnopyranoside	C_36_H_36_O_18_	5.3865	801.1901387	neg
25	Swertiajaponin	C_22_H_22_O_11_	5.3865	507.1147455	neg
26	Flavone base + 4O, C-Hex-FeruloylHex	C_37_H_38_O_19_	5.3968	785.1934000	neg
27	Cynaroside	C_21_H_20_O_11_	5.4181	895.1957948	neg
28	Orientin	C_21_H_20_O_11_	5.4181	447.0937441	neg
29	Camellianin A	C_29_H_32_O_15_	5.4288	619.1679116	neg
30	Bonaniol A	C_25_H_28_O_6_	5.5077	423.1792505	neg
31	Dihydromyricetin	C_15_H_12_O_8_	5.5381	319.0460411	neg
32	Vitexin rhamnoside	C_27_H_30_O_14_	5.5498	577.1569601	neg
33	Alpinone 3-acetate	C_18_H_16_O_6_	5.5732	433.1143198	neg
34	Isovitexin	C_21_H_20_O_10_	5.6571	431.0986599	neg
35	Vitexin	C_21_H_20_O_10_	5.6571	863.2060447	neg
36	Velutin	C_17_H_14_O_6_	5.6697	337.0705801	pos
37	3-Hydroxy-3’,4’,5’-trimethoxyflavone	C_18_H_16_O_6_	5.7143	327.0874000	neg
38	Isoquercitrin	C_21_H_20_O_12_	5.7338	463.0887090	neg
39	7,3’-Dihydroxy-4’-methoxyflavan	C_16_H_16_O_4_	5.8700	317.1035732	neg
40	Scutellarin methylester	C_22_H_20_O_12_	6.0060	475.0886836	neg
41	Pyrroside B	C_26_H_30_O_14_	6.0581	565.1571253	neg
42	baicalin	C_21_H_18_O_11_	6.0804	445.0779809	neg
43	Morin	C_15_H_10_O_7_	6.1011	301.0355157	neg
44	( +)-atechin 5-gallate	C_22_H_18_O_10_	6.1118	441.0830665	neg
45	2’’-O-Rhamnosylicariside II	C_33_H_40_O_14_	6.1506	691.2634695	neg
46	Isorhamnetin-3-O-glucoside	C_22_H_22_O_12_	6.2559	477.1044677	neg
47	Baicalin methyl ester	C_22_H_20_O_11_	6.3433	459.0938391	neg
48	7,4’-Dihydroxy-8-methylflavan	C_16_H_16_O_3_	6.4710	287.1290886	neg
49	Naringenin	C_15_H_12_O_5_	6.5793	273.0758000	pos
50	Liquiritin	C_21_H_22_O_9_	6.6934	417.1191000	neg
51	Cinchonain Ia	C_24_H_20_O_9_	6.7717	497.1096603	neg
52	2’’-O-E-p-Coumaroylafzelin	C_30_H_26_O_12_	6.8871	623.1411859	neg
53	Acacetin	C_16_H_12_O_5_	6.9582	285.0757801	pos
54	Kaempferol-3-O-(6’’-O-cis-coumaryl)glucoside	C_30_H_26_O_13_	7.0174	593.1311226	neg
55	Sagittatoside A	C_33_H_40_O_15_	7.0394	721.2369232	neg
56	Luteolin 7-sulfate	C_15_H_10_O_9_S	7.0509	364.9974465	neg
57	Pinocembrine	C_15_H_12_O_4_	7.1607	255.0663000	neg
58	Quercetin	C_15_H_10_O_7_	7.2791	301.0354000	neg
59	Tupichinol A	C_17_H_18_O_4_	7.3570	331.1189840	neg
60	Eupafolin	C_16_H_12_O_7_	7.4918	317.0656874	pos
61	3-Methoxyflavone	C_16_H_12O3_	7.5205	269.0821102	neg
62	2’,4’-Dihydroxy-7-methoxy-8-prenylflavan	C_21_H_24_O_4_	7.5305	341.1749126	pos
63	8-Prenylnaringenin	C_20_H_20_O_5_	7.5305	341.1384000	pos
64	Tectochrysin	C_16_H_12_O_4_	7.7077	269.0808015	pos
65	Farrerol	C_17_H_16_O_5_	7.9328	301.1070048	pos
66	Kushenol X	C_25_H_28_O_7_	7.9639	485.182379	neg
67	Diosmetin	C_16_H_12_O_6_	7.9724	301.0706075	pos
68	Kaempferide	C_16_H_12_O_6_	7.9752	299.0561000	neg
69	Alpinetin	C_16_H_14_O_4_	8.0490	271.0964265	pos
70	Catechin 3-rhamnoside	C_21_H_24_O_10_	8.0580	401.1231333	pos
71	Gardenin B	C_19_H_18_O_7_	8.1806	381.0966332	pos
72	5-Hydroxy-3’-methoxyflavone	C_16_H_12_O_4_	8.1806	269.0809000	pos
73	7-O-Methylbaicalein	C_16_H_12_O_5_	8.4711	285.0760847	pos
74	Chrysoeriol	C_16_H_12_O_6_	8.6247	301.0707000	pos
75	3,7-Dihydroxy-3’,4’-dimethoxyflavone	C_17_H_14_O_6_	8.7872	315.0863000	pos
76	Sanggenone H	C_20_H_18_O_6_	8.9170	355.1173981	pos
77	Jaceosidin	C_17_H_14_O_7_	8.9170	331.0812344	pos
78	7,4’-Dihydroxy-3’-prenylflavan	C_20_H_22_O_3_	9.0043	343.1904817	pos
79	Neophellamuretin	C_20_H_20_O_6_	9.0987	379.1175345	pos
80	5,7,3’,4’-Tetrahydroxy-3-methoxy-8-geranylflavone	C_26_H_28_O_7_	9.0987	435.1804278	pos
81	Isobavachin	C_20_H_20_O_4_	9.2708	341.1397397	neg
82	Chrysin	C_15_H_10_O_4_	9.2708	253.0506000	neg
83	Chrysosplenetin B	C_19_H_18_O_8_	9.2888	375.1074582	pos
84	Nobiletin	C_21_H_22_O_8_	9.3503	403.1386411	pos
85	Genkwanin	C_16_H_12_O_5_	9.3951	285.0757758	pos
86	2’-Methoxykurarinone	C_27_H_32_O_6_	9.5578	452.2434353	pos
87	Kuwanon C	C_25_H_26_O_6_	9.7721	439.1764693	neg
88	Cyclomorusin	C_25_H_22_O_6_	9.8552	451.1750539	pos
89	Sophoflavescenol	C_21_H_20_O_6_	9.8552	369.1332036	pos
90	Podoverin A	C_21_H_20_O_7_	9.8597	383.1139620	neg
91	Eupatilin	C_18_H_16_O_7_	9.9351	345.0969313	pos
92	Yukovanol	C_20_H_18_O_6_	10.0300	355.1174929	pos
93	Artemetin	C_20_H_20_O_8_	10.2323	389.1229970	pos
94	Morusin	C_25_H_24_O_6_	10.2733	439.1753213	pos
95	Kushenol W	C_21_H_22_O_7_	10.33058	369.1332622	pos
96	Tachrosin	C_23_H_20_O_6_	10.4092	451.1403742	neg
97	(-)-Sophoranone	C_30_H_36_O_4_	10.4211	505.2621458	neg
98	Glabranine	C_20_H_20_O_4_	10.4518	325.1435000	pos
99	Euchrenone A10	C_25_H_26_O_5_	11.3090	451.1768091	neg
100	Kushenol I	C_26_H_30_O_7_	11.7254	437.1959931	pos
101	Apigenin	C_15_H_10_O_5_	15.0555	269.0455000	neg

Flavonoids are ubiquitous plant compounds with rich and diverse biological activities, such as antioxidant^28^, antibacterial^29^, anti-inflammatory^30^, and antiviral actions^31^. Their multiple physiological functions make them widely applicable in medicine, food, and agriculture^32^. We preliminarily identified flavonoids including apigenin, luteolin, isovitexin, naringenin, vitexin, luteoloside, and vitexin-4’’-rhamnoside, which aligns with our earlier findings^33^.

### In vitro antioxidant activity of total flavonoids from leaf material

Oxidative stress is a physiological condition caused by an imbalance between prooxidants and antioxidants, typically manifested as excessive production of reactive species^34^. Under normal circumstances, the production and scavenging of free radicals are maintained in dynamic equilibrium. Disruption of this balance leads to the accumulation of free radicals, which can damage biological molecules such as proteins, DNA, and lipid peroxides, causing diseases such as atherosclerosis^35^, cardiovascular disease^36^, diabetes^37^, tumors^38^, and cancer^39^. Antioxidants can effectively neutralize free radicals, preventing the onset and progression of such diseases.

Because oxidative stress is a complex process, the DPPH, hydroxyl, and ABTS assays were employed to evaluate the antioxidant activity of total flavonoids from *B. papyrifera* leaves.

**Fig. 4:**
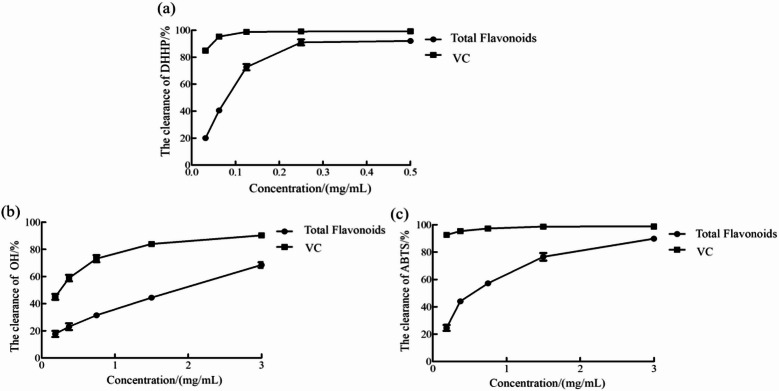
DPPH·(**a**), OH·(**b**), and ABTS (**c**) scavenging ability of total flavonoids.

As shown in Fig. 4a, when the mass concentration of total flavonoid extract from *B. papyrifera* leaves was 0.0125–1 mg/mL, the DPPH radical scavenging rate showed an overall upward trend. The half-maximal inhibitory concentration (IC_50_ value) of total flavonoids against DPPH free radicals was 0.072 mg/mL using software analysis. This activity was substantially higher than that of total flavonoids from *Cedrus deodara pollen* (0.53 mg/mL)^40^ but lower than that of African locust bark extract (0.0074 mg/mL)^41^.

As shown in Fig. 4b, when the concentration of total flavonoids from *B. papyrifera* leaves was 0.1875–3 mg/mL, the hydroxyl radical scavenging rate exhibited an overall increasing trend. The IC_50_ of total flavonoids against hydroxyl radicals was 1.53 mg/mL. Although this activity was substantially higher than that of total flavonoids from *Murraya tetramera* leaves (13.85 mg/mL)^42^, it was lower than that of total flavonoids from *Moxa* (0.185 mg/mL)^43^.

As shown in Fig. 4c, the ABTS radical scavenging rate of the total flavonoid extract from *B. papyrifera* leaves also demonstrated an overall increasing trend within a mass concentration of 0.1875–3 mg/mL. The IC_50_ of total flavonoids against ABTS radicals was 0.48 mg/mL. Although this activity was significantly higher than that of total flavonoids from *Epimedium* (78.259 mg/mL)^44^, it was lower than that of total flavonoids from dandelion (0.306 mg/mL)^45^. Overall, these results demonstrate that total flavonoids from *B. papyrifera* leaves are effective scavengers of DPPH, hydroxyl, and ABTS radicals.

### Determination of the MIC of total flavonoids from leaf material

Bacterial drug resistance has emerged as a critical threat to livestock health and sustainability, making the development of novel antibacterial drugs a major research focus. However, antibiotic development is hampered by long development cycles, high costs, and significant challenges, which have led to growing interest in plant-derived bioactive compounds. Flavonoids, a major class of polyphenolic secondary metabolites, are widely distributed across the plant kingdom^46^. Their broad spectrum of biological activities, including anticancer^47^, antibacterial^48^, and antiviral^49^ properties, combined with advantages such as extensive natural sources, low tendency to induce resistance, and favorable safety profile, have made them the subject of intense research worldwide. Flavonoids exert antibacterial effects through multiple mechanisms, including cell membrane disruption, inhibition of energy metabolism, and impairment of nucleic acid synthesis^50^. They also enhance antibiotic sensitivity. Synergistic interactions between flavonoids and antibiotics improve treatment efficacy against drug-resistant bacteria, highlighting their potential as candidates for developing novel therapeutic agents to combat antimicrobial resistance^51^. Their antibacterial properties, which have been well-documented for their significant inhibitory effects^52^, encompass three primary modes of action: direct bactericidal effects, antibiotic synergism, and reduction of bacterial virulence^53^. Direct inhibition involves multiple mechanisms, including disruption of cell membrane permeability and structure, inhibition of nucleic acid and protein synthesis, suppression or eradication of biofilms, interference with energy metabolism, and inhibition of bacterial motility^54–56^.

To investigate the antimicrobial activity of total flavonoids from *B. papyrifera* leaves, this study examined their effects against *S. aureus*, *E. coli*, and *C. albicans*. The results showed that the MIC values were 25 mg/mL for both *S. aureus* and *E. coli*, and 100 mg/mL for *C. albicans* (Table 5). The inhibitory activity of these flavonoids against *S. aureus* was lower than that reported for total flavonoids from *Dichondra repens*^57^, whereas activity against *E. coli* was higher. Additionally, the anti-*C. albicans* activity was weaker than that of the total flavonoids from *Bidens parviflora*^58^. Previous research has reported that aqueous extracts of *B. papyrifera* leaves inhibit *S. aureus* and *E. coli* with MICs of 125 and 250 mg/mL, respectively^59^, indicating that total flavonoids from *B. papyrifera* possess significantly greater antimicrobial potency than the aqueous extract. Additionally, alcoholic extracts of *B. papyrifera* bark have been reported to inhibit *C. albicans*^60^. In summary, these findings indicate that total flavonoids from *B. papyrifera* leaves are potent antimicrobial agents.

**Table 5:** MIC of total flavonoids to *S. aureus, E. coli*, and *C. albicans.*

Concentration/(mg/mL)	100	50	25	12.5	6.25	Blank comparison	Negative contrast	Positive control
*S. aureus*	−	−—	−	+	+	−	+	−
*E. coli*	−	−	−	+	+	−	+	−
*C. albicans*	−	+	+	+	+	−	+	−

“−” means no colony generation; “ + ” means no colony generation.

Gutiérrez-Venegas et al.^61^ investigated the antibacterial activity of flavonoids such as apigenin, catechin, luteolin, morin, myricetin, naringenin, quercetin, and rutin. Their study demonstrated that these compounds inhibited *Streptococcus pyogenes*, *Enterococcus faecalis*, *E. coli*, and *S. aureus*. Specifically, apigenin was active against *E. coli* and *S. aureus*, whereas catechin inhibited *S. aureus*. Luteolin, naringenin, morin, quercetin, and rutin also inhibited *C. albicans*.

Upon bacterial contact, particularly in the presence of metal ions, many antioxidants (especially polyphenols like tea polyphenols and curcumin) are oxidized, generating large quantities of reactive oxygen species (ROS). This exogenous ROS surge overwhelms the bacterial antioxidant defense system, causing a rapid rise in intracellular ROS. Elevated ROS levels damage cellular components, including membranes, DNA, proteins, and enzymes, leading to bacterial dysfunction and death^62–64^. The strong antioxidant activity of total flavonoids from *B. papyrifera* leaves identified in this study provides a mechanistic basis for their antimicrobial efficacy.

## Conclusion

This study investigated the extraction, antioxidant, and antibacterial properties of total flavonoids from *B. papyrifera* leaves and determined the following optimal extraction conditions: solid-to-liquid ratio of 1:35 g/mL, extraction time of 24.3 min, temperature of 54.7 °C, and ethanol concentration of 83%. The comprehensive UPLC-MS/MS profiling revealed a rich and diverse composition of 101 flavonoids, providing a chemical basis for the observed bioactivities. In vitro antioxidant assays revealed strong radical scavenging activity, with IC_50_ values of 0.072 mg/mL for DPPH, 1.53 mg/mL for hydroxyl, and 0.48 mg/mL for ABTS radicals. Antimicrobial testing revealed MIC values of 25 mg/mL against both *S. aureus* and *E. coli* and 100 mg/mL against *C. albicans*, confirming significant bioactivity. These findings highlight the strong potential of *B. papyrifera* leaf flavonoids as natural antioxidants and antimicrobial agents, with promising applications in the food industry as preservatives, in pharmaceuticals as complementary therapeutic agents, and in veterinary medicine as green feed additives to improve animal health and reduce antibiotic use. However, these findings are solely based on in vitro assays. Further in vivo studies are necessary to evaluate their bioavailability, safety, and efficacy. Future research should also focus on isolation and identification of specific flavonoid compounds, elucidation of their mechanistic studies on their bioactivities, and development of practical formulations for commercial application. Overall, this study provides a scientific basis for the valorization of *B. papyrifera* leaves and contributes to the sustainable utilization of plant resources in functional product development.

## Data Availability

The datasets used and/or analysed during the current study available from the corresponding author on reason-able request.
